# Advanced Composite Materials for Structural Maintenance, Repair, and Control

**DOI:** 10.3390/ma16020743

**Published:** 2023-01-12

**Authors:** Abdul Aabid, Muneer Baig, Meftah Hrairi

**Affiliations:** 1 Department of Engineering Management, College of Engineering, Prince Sultan University, P.O. Box 66833, Riyadh 11586, Saudi Arabia; 2 Department of Mechanical and Aerospace Engineering, International Islamic University Malaysia, P.O. Box 10, Kuala Lumpur 50728, Malaysia

A newly added Special Issue (SI) of the *Materials* journal, titled *"Advanced Composite Materials for Structural Maintenance, Repair, and Control"* focuses on the foundations, characterizations, and applications of several advanced composites. This SI aims to publish reviews and research papers on the most recent scientific and practical studies, including those on product development and the lifecycle analysis of improved advanced composites for engineering applications, particularly aeronautical, mechanical, automotive, material, and structural engineering.

In several engineering applications, defects including delamination, notch, and fracture are unavoidable. These damages are mostly brought on by fatigue and accidents. Structural repair, rather than replacing the entire component, is sometimes the only viable option when the damage to the material is not extensive. Since passive repairs utilize composite materials, they offer enhanced stress transfer mechanisms and joint efficiency. Over the last four decades, bonded composite repair methods by means of various composite material patches, such as carbon-fiber-reinforced polymers, boron-epoxy, carbon-epoxy, and glass-epoxy, have been developed to repair damaged structures. Because they can withstand the imposed stresses at a fraction of the weight of metallic alloys, these materials were appealing to those who dealt with the maintenance, repair, and control of damaged structures. Since then, the usage of composite materials has spread throughout the world, from secondary to primary structures of the aerospace industry, automotive industry, and other fields. New advanced composite materials, repair methods, simulation approaches, and optimization techniques are still being continuously developed with the objective to control structural damage, minimize fracture parameters, enhance cost efficiency, decrease energy consumption, and offer advanced solutions for repair methods and the maintenance of damaged structures.

Bonded composite repair has been discovered to be a successful and productive method for extending the service life of damaged components [1,2,3,4]. The investigations by Rabinovitch et al. [5] used many types of composite patches that are available based on the characteristics of the materials. Additionally, the impact of various patches, including single and double patches, on the lowering of stress intensity factor (SIF) was examined [6,7]. Various sizes and forms of the patches have been developed to show how they affect a damaged structure [8]. Maleki et al. [9] restored an aluminum 2024-T3 plate with a bonded composite patch by analyzing SIF under mixed-mode circumstances, which was different from all the previously published investigations, which were all based on mode-I. Due to its ease of usage and great efficacy in reducing SIF, adhesive-bonded connections are currently utilized more often in structural composites [10]. The structural restoration of damage was investigated in a variety of ways [11,12,13,14,15,16,17]. According to these studies, it is crucial to understand the patch dimensions since a thicker patch leads to a significant reduction in stress intensity, stress concentration, J-integral and many more fracture parameters, but it results in greater weight.

On the other hand, material science and technologies are an important aspect of structural strength. Therefore, this special issue also considered work related on material science and technologies. Experimental techniques and manufacturing processes of lightweight materials and advanced composites can be used to considered with new advanced techniques. In such studies, the effect of manufacturing processes on the quasi-static and dynamic responses of aluminum alloys has been shown using experimental and modelling work [18]. The mechanical properties of lightweight aluminum alloy were also investigated in order to find the strength of the materials [19,20]. Additionally, the mechanical properties of composite materials and their application in a lightweight structure were defined with a few experiments [21]. These works can be done with the newly developed advanced technologies and a soft computing approach.

This special issue aims to present the positive expectations concerning to the repair, maintenance, and control of damaged structures using advanced composite materials. Also, highlights the negative effects brought on by their incorrect or ineffective usage, hoping to offer knowledge and proof in favor of future structural uses that will be effective. Researchers from both academic and industry settings are encouraged to report the outcomes of their experiences, the lessons they’ve learned, and their predictions for this field’s future advancements. Papers from the whole materials and structural community, stressing technical advancements, referencing experimental, numerical, and practical applications, as well as optimizations (such as machine learning, deep learning, the design of experiments, and fuzzy logic), with an emphasis on the predicted and accomplished outcomes, are strongly appreciated.
